# Acceptability and feasibility of leveraging community‐based HIV counselling and testing platforms for same‐day oral PrEP initiation among adolescent girls and young women in Eastern Cape, South Africa

**DOI:** 10.1002/jia2.25968

**Published:** 2022-07-24

**Authors:** Andrew Medina‐Marino, Dana Bezuidenhout, Phuti Ngwepe, Charl Bezuidenhout, Shelley N. Facente, Selly Mabandla, Sybil Hosek, Francesca Little, Connie L. Celum, Linda‐Gail Bekker

**Affiliations:** ^1^ Desmond Tutu HIV Centre University of Cape Town Cape Town South Africa; ^2^ Research Unit Foundation for Professional Development, Buffalo City Metro Eastern Cape Province South Africa; ^3^ Perelman School of Medicine University of Pennsylvania Philadelphia Pennsylvania USA; ^4^ Department of Epidemiology Mailman School of Public Health Columbia University New York City New York USA; ^5^ Department of Statistical Sciences University of Cape Town Cape Town South Africa; ^6^ School of Public Health Boston University Boston Massachusetts USA; ^7^ School of Public Health University of California Berkeley California USA; ^8^ HIV/AIDS STIs and TB Program Buffalo City Metro Health District Eastern Cape Province Department of Health Bhisho South Africa; ^9^ Departments of Psychiatry and Infectious Disease Stroger Hospital of Cook County Chicago Illinois USA; ^10^ Department of Global Health, Medicine and Epidemiology University of Washington Seattle Washington USA

**Keywords:** South Africa, PrEP, community‐based HIV testing, adolescent girls and young women, differentiated care, HIV prevention

## Abstract

**Introduction:**

Community‐based delivery of HIV pre‐exposure prophylaxis (PrEP) to South African adolescent girls and young women's (AGYW) could increase access but needs evaluation. We integrated PrEP services via home‐based services and pop‐up tents into existing community‐based HIV testing services (CB‐HTS) in Eastern Cape Province, South Africa.

**Methods:**

After accessing CB‐HTS via a “pop‐up” tent or home‐based services, HIV‐negative AGYW aged 16–25 years were invited to complete a baseline questionnaire and referred for PrEP services at a community‐based PrEP site co‐located with pop‐up HTS tents. A 30‐day supply of PrEP was dispensed. PrEP uptake, time‐to‐initiation, cohort characteristics and first medication refill within 90 days were measured using descriptive statistics.

**Results:**

Of the 1164 AGYW who tested for HIV, 825 (74.3%) completed a questionnaire and 806 (97.7%) were referred for community‐based PrEP. Of those, 624 (77.4%) presented for PrEP (482/483 [99.8%] from pop‐up HTS and 142/323 [44.0%] from home‐based HTS), of which 603 (96.6%) initiated PrEP. Of those initiating PrEP following home‐based HTS, 59.1% initiated within 0–3 days, 25.6% within 4–14 days and 15.3% took ≥15 days to initiate; 100% of AGYW who used pop‐up HTS initiated PrEP the same day. Among AGWY initiating PrEP, 37.5% had a detectable sexually transmitted infection (STI). Although AGYW reported a low self‐perception of HIV risk, post‐hoc application of HIV risk assessment measures to available data classified most study participants as high risk for HIV acquisition. Cumulatively, 329 (54.6%) AGYW presented for a first medication refill within 90 days of accepting their first bottle of PrEP.

**Conclusions:**

Leveraging CB‐HTS platforms to provide same‐day PrEP initiation and refill services was acceptable to AGYW. A higher proportion of AGYW initiated PrEP when co‐located with CB‐HTS sites compared to those referred following home‐based HTS, suggesting that proximity of CB‐HTS and PrEP services facilitates PrEP uptake among AGYW. The high prevalence of STIs among those initiating PrEP necessitates the integration of STI and HIV prevention programs for AGYW. Eligibility for PrEP initiation should not be required among AHYW in high HIV burden communities. Community‐based service delivery will be crucial to maintaining access to PrEP services during the COVID‐19 pandemic and future health and humanitarian emergencies.

## 1 INTRODUCTION

Adolescent girls and young women (AGYW) make up 10% of the population in sub‐Saharan Africa (SSA), but represent 25% of new HIV infections in the region [1]. In South Africa, the HIV incidence rate among AGYW is four times higher than adolescent boys and young men (ABYM; 2.54% per year vs. 0.55% per year), and AGYW are twice as likely to be living with HIV than ABYM [2, 3]. Unfortunately, despite reductions in global HIV incidence, AGYW in SSA continue to bear a disproportionate burden of HIV infections [4].

HIV oral pre‐exposure prophylaxis (PrEP) had significant efficacy in clinical trials conducted among different population groups [5, 6, 7, 8, 9]. However, young women <25 years had poor efficacy due to poor adherence [10, 11]. Improved PrEP implementation for AGYW will require greater understanding of methods to increase PrEP adherence and persistence among AGYW. The PrEP care cascade provides a framework for understanding PrEP use from a user's perspective, and includes nine steps categorized into three main themes: (1) awareness of PrEP, (2) engaging in PrEP uptake and (3) sustaining adherence and retention in comprehensive PrEP care [12, 13].

Gaps along the PrEP cascade, driven by a variety of behavioural, service delivery, socio‐cultural and other structural challenges, continue to limit the number of people who can benefit from PrEP [14]. The cascade drop‐off that occurs between awareness and uptake has been associated with distance to PrEP services, HIV and antiretroviral stigma, and lack of PrEP sensitization among key supporters [15, 16, 17]. In South Africa, access to PrEP has been predominantly limited to clinic‐based settings [17, 19]. Clinic‐based PrEP services may provide opportunities for service integration; however, these services are fraught with perceived and real barriers for AGYW, including lack of confidentiality, unfriendly clinic staff, long queues, inconvenient operating hours, lack of privacy, a perceived predominant focus on maternal–child health, insufficient AGYW‐friendly services and socio‐cultural biases against AGYW sexual behaviours [18, 19, 20, 21, 22, 23, 24].

Given that HIV testing is the entry point for PrEP service‐delivery, leveraging community‐based HIV testing service (CB‐HTS) platforms to effectively identify and link AGYW to PrEP services should be considered as an intervention for scaling up PrEP. While previous studies have shown effective linkage from CB‐HTS to other HIV prevention services (i.e. voluntary medical male circumcision and family planning) [25, 26, 27, 28], none have integrated nor co‐located same‐day PrEP services into CB‐HTS platforms. Given the needs and barriers to sexual and reproductive health services for AGYW, we investigated the acceptability, feasibility and uptake of same‐day PrEP services by AGYW when integrated into CB‐HTS platforms.

## 2 METHODS

This analysis used baseline cohort and implementation data from the Community PrEP Study (CPS), a randomized controlled trial of a behavioural intervention to improve the effective use of PrEP among AGYW [29]. CPS was conducted in two communities, one urban and one rural, in Buffalo City Metro (BCM) Health District, Eastern Cape Province, South Africa. In 2016, BCM had an estimated population HIV prevalence of 12.4% and incidence of 0.54%; an adult (15+ years) HIV prevalence of 17.1% and incidence of 0.74%; and an AGYW (age 15–24 years) HIV prevalence of 12.8% and incidence of 2.40% [30]. A detailed study protocol and methods has been previously published [29]. Note: exposure to a behavioural intervention only occurred at follow‐up visits and did not impact on CB‐HTS or enrolment activities described below.

### 2.1 HIV testing platforms

Two CB‐HTS modalities (i.e. pop‐up and home‐based testing) were implemented by the study team in the two study communities from 22 October 2018 to 15 November 2019. Pop‐up testings (*n* = 3 per community; 1x fixed; 1x semi‐mobile; 1x portable testing tent) were positioned at key locations in each study community as previously described [29, 31]. Home‐based testing teams systematically visited all households in designated community areas. HIV testing was offered to all community members in accordance with South African national HIV testing guidelines [29, 32].

### 2.2 CB‐HTS, participant recruitment and retention for first medication refill

After completion of post‐test counselling, AGYW aged 16–25 years with negative HIV test results were screened using a standardized script read in English or IsiXhosa (Table S1: Script 1). Eligible individuals (e.g., self‐identifying as a woman, aged 16–25 years, HIV‐negative test) were then read a second standardized script inviting them to complete a baseline questionnaire (Table S1: Script 2).

Consenting participants were then administered a baseline questionnaire, which included a short 5 minute PrEP informational video featuring AGYW from similar communities (https://www.youtube.com/watch?v=rHkQq–anmo) [33]. Upon completion, participants interested in learning more about PrEP were referred to fixed service sites in each study community where they learned more about PrEP services.

Interested participants who presented to the PrEP service sites were provided additional information about PrEP. Those interested in initiating PrEP were further assessed for study eligibility. Inclusion criteria for study participation and PrEP initiation were self‐reported: (1) age 16–25 years old, (2) self‐identified as female, (3) residing in one of the study communities, (4) HIV negative, (5) fluent in English or IsiXhosa, (6) no plan to move outside of the study community for 12 months and (7) willing and able to provide informed consent. Participants were excluded if they self‐reported: (1) being pregnant, (2) breastfeeding, (3) participating in another HIV prevention study, (4) using post‐exposure prophylaxis or (5) taking tuberculosis treatment. Due to social desirability bias, structural risk factors and the dynamic nature of HIV risk among South African AGYW, prior sexual activity was not used as inclusion/exclusion criterion [3, 34, 35].

Eligible participants were fully briefed on the study, consented and asked to provide urine and blood specimens [29]. Non‐pregnant AGYW were then provided a 30‐day supply of DIDIVIR (CIPLA generic for Emtricitabine 200 mg/Tenofovir Disoproxil Fumarate 300 mg). All participants were asked to return for monthly PrEP refills.

Given the recommendation that young people on PrEP may benefit from more frequent visits to support adherence [36] and to encourage return for a first medication refill, participants were invited for an orientation visit 2 weeks post‐enrolment to meet with their adherence counsellor, review test results with a study nurse, assess side effects from PrEP and receive the first of three hepatitis B injections if indicated.

### 2.3 Data and specimen collection

A baseline audio computer‐assisted self‐interviewing questionnaire, administered on a table computer following HTS post‐test counselling, collected socio‐demographics, behavioural and clinical histories; HIV knowledge, attitudes and practice; and a self‐reported HIV risk survey. Implementation indicators for study monitoring were collected using REDCap. Upon study consent to initiate PrEP, urine was collected for pregnancy testing, vaginal swabs were collected to test for sexually transmitted infections (STIs) and blood specimens were collected for hepatitis B antigen, syphilis and creatinine clearance [29].

### 2.4 Data analysis

The distributions of individual‐level characteristics were calculated using frequency and proportions for discrete variables and medians with interquartile range (IQR) for continuous measures. Significance testing was not conducted because the study was not powered to measure differences in testing modalities. Participant retention for the first medication collection was calculated using the number of participants who attended the visit divided by the study sample size.

To assess factors associated with presentation to a PrEP initiation site following home‐based testing and referral, we conducted a multivariable logistic regression model. This analysis was restricted to the home‐based modality because there was near‐universal same‐day initiation from the pop‐up testing modality (Figure 2). Variable selection for our model was based on a directed acyclic graph, which included factors hypothesized or documented in the literature to be associated with HIV prevention behaviours and PrEP uptake [37]. First, crude logistic regression was used to estimate associations between presentation and each factor individually. Due to the exploratory nature of this analysis, factors whose *p*‐value was ≤ 0.25 or suggestive in the literature to be significant were included together in a multivariable model to adjust for potential confounding.

Using descriptive statistics, we compared the overall HIV risk profile of our study cohort to that of other PrEP demonstration study cohorts by applying previously used HIV risk assessment measures in a post‐hoc analysis [10, 38, 39, 40]; of note, we had not collected all variables assessed by these measures. The ICAP risk assessment tool was developed to support the implementation of PrEP; training curriculum and tools were designed to enable clinical providers to attain the skills required to provide PrEP to appropriate candidates [41]. For the ICAP tool to screen for substantial risk of HIV [38], we applied the “sexual activity in a high‐prevalence HIV population” criteria plus condomless sex with more than one partner or a history of STIs via self‐report, lab test or syndromic screening. The Liverpool VCT, Care and Treatment (LVCT) Health risk assessment tool was developed to identify young women at risk for HIV for the Introducing PrEP into HIV Combination Prevention demonstration project [39]. For the LVCT tool [39], we collected and used five of the seven data points, including current age, age at first sexual interaction, condom use, STI history and number of sexual partners all in the last 3 months; we did not collect HIV status of sexual partners and pregnancy was a study exclusion criterion. For the VOICE risk assessment score [40, 42], we collected and used six of the seven data points, including age, married or living with partner, partner provides financial/material support, primary sex partner has other partners, any curable STI and alcohol use; we did not collect HSV‐2 serological data. Of note, the VOICE risk assessment score was derived from women who participated in the MTN 033/VOICE study, and further assessed using data from women enrolled in MTN 020/ASPIRE [10, 43]. It was validated to predict 1‐year risk of HIV acquisition among African women in settings with generalized HIV epidemics, including South Africa; a risk score of ≥3 correlated with an HIV incidence of >3% per year. VOICE was developed to inform targeted scale‐up of HIV prevention programs, including PrEP, for women in Eastern and Southern Africa.

All analyses were conducted using STATA 13.1 software (StataCorp, College Station, TX, USA).

### 2.5 Ethics approval

Ethics approval was obtained from the University of Cape Town Human Research Ethics Committee (Ref: 289/2018) with permission provided by the Eastern Cape Provincial Department of Health research committee and the BCM District Department of Health.

## 3 RESULTS

**Figure 1:**
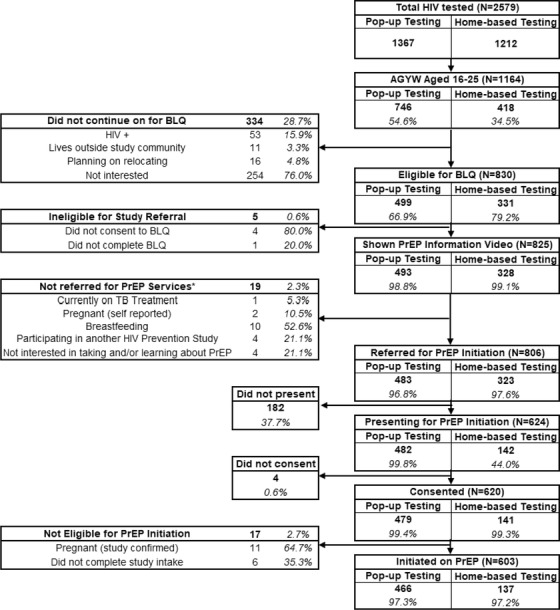
Participant enrolment flow diagram.

A total of 1164 AGYW were tested for HIV; 746 (64.1%) via pop‐up testing and 418 (35.9%) via home‐based testing (Figure 1). Of those, 1111 (95.4%) were read Script 1; 53 (4.55%) tested positive for HIV and were not eligible for further consideration. Of those read Script 1, 254 (22.9%) immediately expressed they did not want to participate in any survey, 11 (1%) were not eligible because they lived outside the study communities and 16 (1.4%) reported they planned to relocate in the next 12 months.

**Figure 2:**
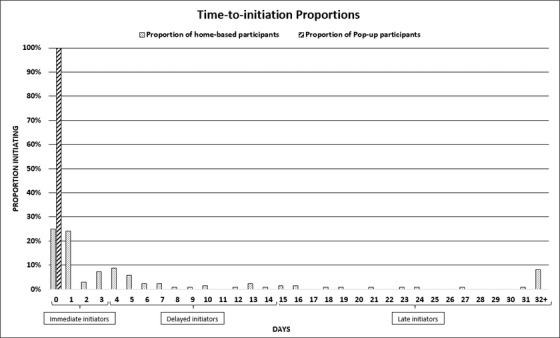
Time‐to‐initiation proportions.

Of the 830 (74.7%) AGYW read Script 2, 826/830 (99.5%) consented to take the baseline questionnaire, 825/830 (99.4%) completed the questionnaire and 806/830 (97.1%) were referred for PrEP initiation services. Of those referred, 624/806 (77.4%) presented to a community‐based PrEP initiation site; 482/483 (99.8%) presented from pop‐up testing sites and 142/323 (44.0%) presented from home‐based testing. Of those who presented, 620/624 (99.4%) provided consent and 603/620 (97.3%) initiated PrEP; 466/479 (97.3%) from pop‐up testing and 137/141 (97.2%) AGYW home‐based testing (Figure 1). Hundred percent of eligible AGYW from the pop‐up testing modality initiated PrEP the same day as introduction. In comparison, 59.1% of home‐based testers initiated within 0–3 days, 25.6% initiated within 4–14 days and 15.3% initiated within 15+ days from introduction (Figure 2). There were no significant socio‐demographic or behavioural factors that influenced the presentation to a PrEP initiation site from home‐based referrals; notably, only participants from home‐based referrals were analysed, as 97.3% of participants referred from a mobile site presented for PrEP initiation (Table S2).

The median age of study participants was 18 years (IQR: 4), with the majority attending school and reporting their average monthly income to be less than R1000 (∼$57.80) (Table 1). Previous HIV testing was self‐reported by 562 (93.2%) participants, of which 315 (52.2%) had tested more than three times, and 360 (59.7%) reported testing for HIV within the last 6 months. The median age of sexual debut was 16 years (IQR: 2), with 385 (63.8%) participants reported ever engaging in sexual intercourse, and 284 (47.1%) reporting sexual intercourse in the past month; only 120 (20.0%) participants reported always using a condom. The majority of participants (*n* = 335; 55.6%) reported having a primary sex partner. Among those who reported having a primary sex partner, 240 (71.6%) stated that they either knew or were unsure whether their primary sex partner had other sex partners. Though 570 (94.5%) participants had discussed HIV prevention strategies, only 250 (41.5%) had heard of PrEP before our study.

**Table 1:** Characteristics of study participants stratified by testing modality

Characteristics		Total (603; 100.0%)	Pop‐up testing (466; 77.3%)	Home‐based testing (137; 22.7%)
Socio‐demographic characteristics				
Age (median; IQR)		18 (4)	18 (5)	20 (5)
Current school attendance	Yes	407 (67.5)	334 (71.7)	73 (53.3)
	No	196 (32.5)	132 (28.3)	64 (46.7)
Level of education completed	No formal schooling	35 (5.8)	28 (6.0)	7 (5.1)
	High school	458 (76.0)	355 (76.2)	103 (75.2)
	Above high school and other	105 (17.4)	80 (17.2)	25 (18.2)
Monthly income	≤R1000	444 (73.6)	333 (71.5)	111 (81.0)
	R1001–R4999	47 (7.8)	37 (7.9)	10 (7.3)
	≥R5000	24 (4.0)	21 (4.5)	3 (2.2)
Household size (median; IQR)		5 (3.5)	5 [3]	4 [3]
Location	Urban	303 (50.2)	260 (55.8)	43 (31.4)
	Rural	300 (49.8)	206 (44.2)	94 (68.6)
Behavioural characteristics				
HIV test	Yes	562 (93.2)	431 (92.5)	131 (95.6)
	No	32 (5.3)	27 (5.8)	5 (3.7)
	Don't know	4 (0.7)	4 (0.9)	0 (0.0)
Number of HIV tests^a^	0 times	9 (1.5)	8 (1.7)	1 (0.73)
	1–2 times	234 (38.8)	184 (39.5)	50 (36.5)
	3–4 times	169 (28.0)	135 (29.0)	34 (24.8)
	5 or more times	146 (24.2)	101 (21.7)	45 (32.8)
Time since last test^a^	Less than 3 months ago	254 (42.1)	183 (42.5)	71 (54.2)
	3–6 months ago	106 (17.5)	82 (19.0)	24 (18.3)
	7–12 months ago	59 (9.8)	49 (11.4)	10 (7.6)
	More than 12 months ago	37 (6.1)	28 (6.5)	9 (6.9)
	Cannot recall	86 (14.3)	71 (16.5)	15 (11.5)
Alcohol use	Yes	333 (55.2)	261 (56.0)	72 (52.6)
	No	260 (43.1)	195 (41.9)	65 (47.4)
Drug use	Yes	39 (6.5)	35 (7.5)	4 (2.9)
	No	557 (92.4)	425 (91.2)	132 (96.4)
Ever had sexual intercourse	Yes	385 (63.8)	302 (64.8)	83 (60.6)
	No	179 (29.7)	130 (27.9)	49 (35.8)
Age at sexual debut (median; IQR)		16 (2)	16 (2)	17 (2)
Sexuality^b^	Opposite sex	398 (66.0)	316 (67.8)	82 (59.9)
	Bisexual	8 (1.3)	6 (1.3)	2 (1.5)
	Same sex	9 (1.5)	5 (1.1)	4 (2.9)
Time since last sexual intercourse^b^	Within past week	198 (32.8)	158 (33.9)	40 (29.2)
	Within past month	86 (14.3)	69 (14.8)	17 (12.4)
	1–6 months ago	80 (13.3)	61 (13.1)	19 (13.9)
	7–12 months ago	9 (1.5)	8 (1.7)	1 (0.7)
	More than a year ago	32 (5.3)	22 (4.7)	10 (7.3)
Frequency of condom use^b^	All the time	120 (20.0)	92 (19.7)	28 (20.4)
	Some of the time	230 (38.1)	187 (40.1)	43 (21.4)
	Never	66 (10.9)	49 (10.5)	17 (12.4)
Ever received incentive for sex^c^	Yes	120 (19.9)	94 (20.2)	26 (19.0)
	No	54 (9.0)	48 (10.3)	6 (4.4)
	Don't know	110 (18.2)	82 (17.6)	28 (20.4)
Ever had sex with a partner living with HIV^b^	Yes	4 (0.7)	4 (0.9)	0 (0.0)
	No	356 (59.0)	281 (60.3)	75 (54.7)
	Don't know	61 (10.1)	48 (10.3)	13 (9.5)
Primary sex partner	Yes	335 (55.6)	260 (55.8)	75 (54.7)
	No	240 (39.8)	187 (40.1)	53 (38.7)
Primary sex partner has other sex partners^d^	Yes, I know	53 (8.8)	41 (8.8)	12 (8.8)
	Yes, I suspect	90 (14.9)	68 (14.6)	22 (16.1)
	Unknown	124 (20.6)	93 (20.0)	31 (22.6)
	No	94 (15.6)	75 (16.1)	19 (13.9)
	Don't know	2 (0.3)	2 (0.4)	0 (0.0)
Clinical characteristics				
Depression (PHQ9)	Normal (0–4)	229 (38.0)	171 (36.7)	58 (42.3)
	Mild (5–9)	226 (37.5)	177 (38.0)	49 (35.8)
	Moderate (10–14)	113 (18.7)	92 (19.7)	21 (15.3)
	Moderately severe (15–19)	27 (4.5)	21 (4.5)	6 (4.4)
	Severe (20–27)	8 (1.3)	5 (1.1)	3 (2.2)
Creatinine clearance	Positive (<60)	11 (1.8)	10 (2.2)	1 (0.7)
	Negative (>60)	582 (96.5)	447 (95.9)	135 (98.6)
	Missing	10 (1.7)	9 (1.9)	1 (0.7)
Hepatitis B antigen (HBsAg)	Positive	2 (0.3)	1 (0.2)	1 (0.7)
	Negative	579 (96.7)	448 (97.6)	131 (93.6)
	Invalid	17 (2.8)	9 (2.0)	8 (5.7)
	Missing	1 (0.2)	1 (0.2)	0 (0.0)
Ever diagnosed with STI	Yes	113 (18.7)	84 (18.0)	29 (21.2)
	No	480 (79.6)	373 (80.0)	107 (78.1)
STI symptoms^g^	Yes	209 (34.7)	170 (36.5)	39 (28.5)
	No	385 (63.8)	288 (61.8)	97 (70.8)
STI—any	Yes	225 (37.5)	181 (39.1)	44 (32.4)
	No	366 (61.1)	277 (59.8)	89 (65.4)
STI^e^—by infection	CT infection	182 (30.8)	141 (30.8)	41 (30.8)
	NG infection	59 (10.0)	48 (10.5)	11 (8.3)
	TV infection	42 (7.1)	35 (7.6)	7 (5.3)
Syphilis test	Positive	2 (0.3)	2 (0.4)	0 (0.0)
	Negative	600 (99.0)	459 (98.9)	141 (100.0)
	Missing	3 (0.7)	3 (0.7)	0 (0.0)
Knowledge, attitudes, practice and self‐perceived risk of HIV				
Positive STI by self‐report sexual activity^f^	Had sex	160 (73.4)	N/A	
	Never had sex	58 (26.6)	N/A	
Ever discussed HIV prevention with anyone	Yes	570 (94.5)	437 (93.8)	133 (97.1)
	No	14 (2.3)	13 (2.8)	1 (0.7)
Ever heard of PrEP	Yes	250 (41.5)	188 (40.3)	62 (45.3)
	No	347 (57.5)	273 (58.6)	74 (54.0)
Knowledge of HIV risk (median; IQR) Range: 0–7		3 (3)	3 (3)	2 (4)
Self‐perceived HIV risk (median; IQR) Range: 0–10		1 (4)	1 (4)	1 (3)

^a^ N/A for 41/57 participants who have not had an HIV test.

^b^ N/A for 179 participants who reported never having sex.

^c^ Sex incentives included: money, alcohol/drugs, clothes, airtime, accommodation, transport, better academic grades, school fees and food.

^d^ N/A for 240 participants who do not have primary sex partner.

^e^ Denominator is those with a valid test result.

^f^ Denominator is those with a positive STI test result who did not skip the sexual activity question.

^g^ Symptom screening performed as per South African national guidelines for syndromic management of STIs.

Prior to study enrolment, 113 (18.7%) participants reported ever being treated for an STI. At the time of enrolment, 227 (37.6%) participants had a positive STI test result, of which 134 (59.0%) were asymptomatic. *Chlamydia trachomatis* (*CT, n* = 182; 30.6%) accounted for the highest burden STI, followed by *Neisseria gonorrhoeae* (*NG, n* = 59; 10.0%), *Trichomonas vaginalis* (*TV, n* = 42; 7.1%) and syphilis (*n* = 2, 0.3%) (Table 1). Analysis of participant sexual behaviours and STI test results revealed that of the 218 AGYW with positive STI results who responded to the question about sexual activity, 58 (26.6%) reported never having had sex (Table 1). Furthermore, of 120 participants who reported always using a condom, 41 (34.2%) had a positive STI test result (data not shown).

On a scale of 0 to 10 (0 = no risk; 10 = extreme risk) for self‐perceived risk of HIV infection, the median score self‐reported by participants was 1 (IQR: 4; range 0–10). When computing our study population's risk profile from more in‐depth risk assessments used during previous PrEP studies [10, 39], 64.2–88.5% of our study population would be considered at high risk of contracting HIV (Table S3).

Implementation indicators are shown in Table S4. The number of AGYW needed to test to identify one AGYW interested in PrEP was 1.51 for pop‐up testing services and 1.27 for home‐based testing services. To refer one AGYW for PrEP services, 1.54 AGYW needed to be tested at a pop‐up testing site, while only 1.29 AGYW needed to be tested through home‐based testing services. Finally, in order to initiate one AGYW on PrEP, 1.60 AGYW needed to be tested from a pop‐up testing site, while 3.05 AGYW needed to be tested via home‐based testing services (Table S4).

Cumulative retention for participants’ first medication collection is shown in Table 2. Within the first 30 days of study participation, 229 (38.0%) participants presented for their first medication refill (38.2% of pop‐up testing participants and 36.5%% of home‐based testing participants). Within 60 days of study participation, an additional 78 participants presented for their first medication refill, increasing the cumulative presentation to 50.9% (51.5% of pop‐up testing participants and 48.9% of home‐based testing participants). Within 90 days of study participation, an additional 22 participants presented for their first medication refill, increasing the cumulative presentation to 54.6% (55.2% of pop‐up testing participants and 52.6% of home‐based testing participants).

**Table 2:** Cumulative participant retention for first medication collection at 30, 60 and 90 days

Retention						
Time point	Pop‐up testing		Home‐based testing		Total	
	*# Attended*	*%*	*# Attended*	*%*	*# Attended*	*%*
First refill within 30 days	178/466	38.2%	50/137	36.5%	229/603	38.0%
First refill within 60 days	240/466	51.5%	67/137	48.9%	307/603	50.9%
First refill within 90 days	257/466	55.2%	72/137	52.6%	329/603	54.6%

## 4 DISCUSSION

Our study sought to reduce barriers to clinic‐based PrEP services for South African AGYW. AGYW in South Africa continue to be at high risk for HIV acquisition [3], with a significant proportion of our participants self‐reporting infrequent condom use, transactional sex and not knowing their sexual partner's HIV status. While our study purposefully did not apply HIV risk measures to determine eligibility for initiating PrEP, our post‐hoc analysis found that a significant proportion of study participants were at high risk of acquiring HIV speaking to the disconnect between AGYW's self‐perceived HIV risk and actual HIV risk. Unequal gender dynamics associated with transactional sex and sexual relationships with older men likely contribute to low levels of condom use for many AGYW [44, 45, 46]. Furthermore, the large proportion of study participants who reported receiving incentives for sex likely indicates limited access to financial resources. Given these findings, increased access to community‐based PrEP services may better support AGYW to mitigate their risk of HIV infection. High interest in and uptake of PrEP among study participants indicate substantial demand for such services.

AGYW face numerous barriers in obtaining clinic‐based sexual and reproductive health services, including perceived or real issues pertaining to confidentiality [47, 48], unfriendly clinic staff [48, 49, 50], physical access to clinics [51, 52] and social acceptability of sexual activities [53]. We found that co‐location of pop‐up HTS and community‐based PrEP services resulted in a higher rate of presentation for PrEP services compared to referral following home‐based HTS, suggesting that co‐located CB‐HTS and PrEP services may be preferable to AGYW. Interestingly, HTS modality was not associated with different rates of PrEP initiation nor return for their first medication refill within 90 days (Table S4); however, our previous qualitative assessment identified barriers and facilitators to immediate and delayed presentation to our community‐based PrEP service sites [54].

Given the cost and logistics associated with establishing non‐clinic, community‐based PrEP services (e.g. operational and fixed costs akin to a community pharmacy dispensing site), referrals for clinic‐based PrEP services are likely to be more feasible and cost‐effective for large‐scale HIV prevention programs, especially due to the large number of existing non‐government organization and community health worker programs already providing community‐based health services in South Africa [33, 34, 35, 36, 37, 38, 39, 40, 41, 42, 43, 44, 45, 46, 47, 48, 49, 50, 51, 52, 53, 54, 55, 56, 57]. However, leveraging community‐based platforms for other HIV prevention services, such as injectable PrEP, will be strongly influenced by the cold‐chain requirements of the injectable formulation. This said, it may be highly amenable to home‐based delivery.

Although a growing number of studies and programs are offering same‐day PrEP initiation, few have provided AGYW access to PrEP via non‐clinical settings [58, 59, 60, 61]. Previous studies and population‐based programs have shown same‐day initiation rates between 77.0% and 90.8% [59, 60, 62]. In contrast, among pop‐up testers offered same‐day initiation in our study, 100% initiated PrEP that day; 59.1% of those referred from home‐based HTS presented for PrEP within 3 days. Of the 603 participants who initiated PrEP, only 11 (1.8%) had abnormal creatinine clearance results at initiation, further supporting the South African HIV Clinicians Society guidelines to assess creatinine during or after PrEP initiation [63]. Together, these findings support the co‐location of CB‐HTS and PrEP services offering same‐day initiation for AGYW.

PrEP scale‐up in South Africa provides an additional opportunity to integrate STI services with HIV prevention. Recent studies in SSA have shown extremely high prevalence and incidence of curable STIs (*CT*, *NG* and *TV*) [64, 65, 66]. Almost 90% of those young women and 59.0% of participants in this study were asymptomatic and would have been missed by WHO's syndromic management approach. It is essential to identify improved STI control strategies that are effective, affordable and put STI prevention into AGYW's hands [67]. Acceptable and feasible ways to identify and treat sexual partners are also needed to significantly improve reproductive health outcomes and impact of PrEP.

Of note, our implementation of CB‐HTS platforms and concomitant recruitment of participants was concluded prior to the global occurrence of COVID‐19 [68], with all participants due for a first medication pick‐up prior to 16 February 2020. This was before both the first case of COVID‐19 was identified in South Africa and the government's implementation of public health measures, including the national lock‐down [69, 70]. Consequently, COVID‐19 had no effect or impact on the implementation of our CB‐HTS services, recruitment activities or the ability of participants to pick up their first medication refill within 90 days of PrEP initiation. However, a number of studies have shown wide‐spread disruptions to clinic‐based HIV and PrEP services during the COVID‐19 pandemic [71, 72, 73]. Community‐based service delivery has been shown to improve access to HIV services during previous health and conflict emergencies [74], and when compared to accessing clinic‐based services even during non‐emergency times [75]. Due to historic over‐crowding of primary health clinics, we and others continue to suggest that community‐based service delivery is crucial both to minimize COVID transmission in primary health clinics, including to healthcare workers, and to maintain access to PrEP and other HIV care and treatment services during and after the COVID‐19 pandemic [71, 72, 73, 74, 76, 77, 78]. However, the effectiveness and sustainability of such community‐based service delivery platforms during and after COVID‐19 will depend on the mobilization of health resources.

A significant strength of this study was the ability to leverage widely implemented CB‐HTS to assess the acceptability and feasibility of PrEP services provision via non‐clinic‐based platforms. Towards this, the inclusion of two different CB‐HTS platforms was also a strength, as they provided comparative implementation data to inform potential future scale‐up activities. This study also has some limitations. First, nearly a third of AGYW with a negative HIV test result immediately declined to hear anything about the study. As such, a rather large number of AGYW potentially eligible for PrEP never had the chance to learn more about PrEP as an HIV prevention method. Notably, refusal occurred after being read Script 1, which did not include any mention of PrEP; thus, we cannot conclude that refusal had anything to do with lack of interest in PrEP or HIV prevention services. Second, our study was not powered to compare PrEP uptake or persistence between the community‐based recruitment modalities. Although observational comparisons were noted, a rigorous evaluation should be performed to determine the most cost‐effective delivery model. Third, this study did not have a clinic‐based referral comparison to determine how uptake rates may differ between referrals for community‐ versus clinic‐based PrEP services. Although we observed high presentation rates for community‐based PrEP services, we cannot conclude that this would be significantly different from the presentation rates for clinic‐based PrEP services.

## 5 CONCLUSIONS

Leveraging CB‐HTS platforms to provide same‐day PrEP initiation services is an important addition to existing HIV prevention and testing behaviours of AGYW. CB‐HTS was shown to be acceptable and available for AGYW [31]. Despite high HIV incidence in this subgroup, PrEP uptake and adherence has remained low [10]. We report that providing community‐based PrEP services directly increases supply‐side aspects of the PrEP cascade for AGYW, and leveraging community platforms to increase the knowledge of PrEP services may also increase the demand for PrEP services in this group. Integrating community‐based PrEP promotion and services with CB‐HTS may ultimately lead to substantial improvement in access and impact of PrEP for AGYW in SSA, helping to reduce HIV‐related health inequities currently seen in this population.

## COMPETING INTERESTS

SNF reports receipt of consulting fees from Gilead Sciences, Inc. for studies unrelated to this current work. Gilead had no influence over the data collection, analysis or reporting of this study.

## AUTHORS’ CONTRIBUTIONS

AMM and LGB developed the original study concept and design. AMM, LGB, SH, CLC and SNF contributed to the development of the funded NIH grant submission. AMM and CB developed the study concept, design and funding proposal to the Bill and Melinda Gates Foundation via the South African National HIV Think Tank. AMM, CB, DB and PN oversaw all aspects of study implementation. AMM, DB, CB and PN developed the detailed protocol implementation plan and oversaw all study activities and staff. DB, PN and FL were responsible for data management and analysis. AMM and DB led the writing of the manuscript, and SNF supported manuscript revision. All authors approved the final version of this manuscript.

## FUNDING

This research is funded by the National Institute of Mental Health (NIMH) of the U.S. National Institutes of Health under award number R01MH114648 to AMM and LGB. Complementary funding was provided by the Bill and Melinda Gates Foundation though the South African National HIV Think Tank to AMM. The funders had no role in the study design, data collection and analysis, nor will they have any role in manuscript preparations of publication decisions.

## DISCLAMER

The authors wrote the manuscript and had final responsibility for the decision to submit for publication. The funder had no role in design, data collection, analysis, interpretation or writing of the report. The authors alone are responsible for the views expressed in this article and they do not necessarily represent the views, decisions or policies of the donor.

## Supporting information


**Table S1**: Enrolment Scripts.

**Table S2**: Socio‐demographic and behavioural factors associated with presentation for PrEP services among AGYW receiving home‐based HTS.

**Table S3**: Application of risk assessment tools to the community PrEP study participants.

**Table S4**: Implementation indicators.

## Data Availability

Data may be available upon request.
