# Supplementary material for: Acceptability and feasibility of leveraging community‐based HIV counselling and testing platforms for same‐day oral PrEP initiation among adolescent girls and young women in Eastern Cape, South Africa
Source: J Int AIDS Soc. 2022 Jul 24;25(7):e25968. doi: 10.1002/jia2.25968 (PMC9309460; doi:10.1002/jia2.25968)
Supplement: Supplementary file 1 — Table S1: Enrolment Scripts. [file JIA2-25-e25968-s003.docx]

**Supplemental Table 1:** Enrollment Scripts

| **Script 1:** |
| --- |
| *“We are conducting a community survey in your area today. Before we can ask you to participate in this survey we need to check whether you are eligible to take the survey. Would you mind if I ask you a quick couple of questions?”* |
| **Script 2:** |
| *“As you know, HIV/AIDS is a big problem facing millions of South Africans every year. We are currently doing a survey in your community to try and find out how young women, like yourself, are being affected by risks associated with HIV/AIDS. The survey that we are doing at the moment has questions related to an individual’s demographics as well as other topics including socioeconomic information, sexual behaviours, social support and their knowledge regarding PrEP medication. The information that we gather from this survey will help us identify ways of helping young women, like yourself, stay protected from getting infected with HIV/AIDS. If you are willing to complete this survey today we will use a consent form to obtain proof of your willingness to participate in this research activity.”* |
