# Supplementary material for: Acceptability and feasibility of leveraging community‐based HIV counselling and testing platforms for same‐day oral PrEP initiation among adolescent girls and young women in Eastern Cape, South Africa
Source: J Int AIDS Soc. 2022 Jul 24;25(7):e25968. doi: 10.1002/jia2.25968 (PMC9309460; doi:10.1002/jia2.25968)
Supplement: Supplementary file 2 — Table S2: Socio‐demographic and behavioural factors associated with presentation for PrEP services among AGYW receiving home‐based HTS. [file JIA2-25-e25968-s002.docx]

**Supplemental Table 2:** Sociodemographic and behavioural factors associated with presentation for PrEP services among AGYW receiving home-based HTS

| Factors Influencing Presentation of Home-based Testers | | | | | |
| --- | --- | --- | --- | --- | --- |
| Variable | | Crude analysis | | Multivariable analysis | |
|  |  | Odds Ratio (95% *CI*) | *P-value* | Odds Ratio (95% *CI*) | *P-value* |
| Location | Rural | 1.36 (0.85, 2.16) | 0.20 | 1.34 (0.60, 3.02) | 0.48 |
|  | Urban | REF | | REF | |
| Age | | 1.00 (0.92, 1.08) | 0.99 |  |  |
| Current School Attendance | Yes | 0.88 (0.57, 1.37) | 0.58 |  |  |
|  | No | REF | |  |  |
| Level of Education Completed | No formal schooling | 1.59 (0.60, 4.27) | 0.45 |  |  |
|  | Above high school/Other | 1.04 (0.59, 1.85) | 0.89 |  |  |
|  | High school | REF | |  |  |
| Household Size | | 0.92 (0.84, 1.01) | 0.08 | 0.95 (0.81, 1.14) | 0.60 |
| HIV Test | Yes | 1.98 (0.69, 5.70) | 0.20 | 0.71 (0.14, 3.65) | 0.68 |
|  | No | REF | | REF | |
| Alcohol Use | Yes | 0.94 (0.61, 1.47) | 0.80 |  |  |
|  | No | REF | |  |  |
| Ever had sexual intercourse | Yes | 0.95 (0.60, 1.52) | 0.85 |  |  |
|  | No | REF | |  |  |
| Age at Sexual Debut | | 0.98 (0.83, 1.15) | 0.77 |  |  |
| Frequency of condom use | All the time | 0.74 (0.32, 1.70) | 0.47 |  |  |
|  | Some of the time | 0.66 (0.30, 1.44) | 0.30 |  |  |
|  | Never | REF | |  | |
| Ever received incentive for sex | Yes | 1.88 (0.92, 3.87) | 0.08 | 1.81 (0.85, 3.87) | 0.13 |
|  | No | REF | |  |  |
| Primary sex partner | Yes | 1.38 (0.87, 2.19) | 0.17 | 1.27 (0.53, 3.09) | 0.59 |
|  | No | REF | |  |  |
| Self-perceived HIV risk | | 1.00 (0.92, 1.09) | 0.95 |  |  |
