# Supplementary material for: Acceptability and feasibility of leveraging community‐based HIV counselling and testing platforms for same‐day oral PrEP initiation among adolescent girls and young women in Eastern Cape, South Africa
Source: J Int AIDS Soc. 2022 Jul 24;25(7):e25968. doi: 10.1002/jia2.25968 (PMC9309460; doi:10.1002/jia2.25968)
Supplement: Supplementary file 3 — Table S3: Application of risk assessment tools to the community PrEP study participants. [file JIA2-25-e25968-s001.docx]

| Supplemental Table 3: Application of risk assessment tools to the community prep study participants | | | | |
| --- | --- | --- | --- | --- |
| Community PrEP Study Population | | | | |
| Study Tool | **Risk**  **Category** | **# of study participants** | | |
|  |  | **Total** | **Pop-up Testing** | **Home-based Testing** |
| VOICE Scale(19) | <5 (low risk) | 69 (11.5%) | 53 (11.4%) | 16 (11.8%) |
|  | ≥5 (high risk) | 530 (88.5%) | 410 (88.6%) | 120 (88.2%) |
| ICAP Scale(40) | Low risk | 216 (35.8%) | 162 (34.8%) | 54 (39.4%) |
|  | High risk | 387 (64.2%) | 304 (65.2%) | 83 (60.6%) |
| LVCT Scale(41) | <2 (low risk) | 103 (17.2%) | 66 (14.3%) | 37 (27.2%) |
|  | ≥2 (high risk) | 496 (82.8%) | 397 (85.7%) | 99 (72.8%) |
