# Supplementary material for: Acceptability and feasibility of leveraging community‐based HIV counselling and testing platforms for same‐day oral PrEP initiation among adolescent girls and young women in Eastern Cape, South Africa
Source: J Int AIDS Soc. 2022 Jul 24;25(7):e25968. doi: 10.1002/jia2.25968 (PMC9309460; doi:10.1002/jia2.25968)
Supplement: Supplementary file 4 — Table S4: Implementation indicators. [file JIA2-25-e25968-s004.docx]

**Supplemental Table 4:** Implementation Indicators

| **Indicator** | **Modality** | |
| --- | --- | --- |
|  | **Pop-up Testing** | **Home-based Testing** |
| **HCT Testing Indicators** | | |
| # needed to test to find one AGYW | 1.83 | 2.90 |
| # AGYW needed to test to find one interested in taking PrEP | 1.51 | 1.27 |
| # AGYW needed to test to refer one for PrEP services | 1.54 | 1.29 |
| # AGYW needed to test to initiate one on PrEP | 1.60 | 3.05 |
| **Household Indicators (home-based testing only)** | | |
| # households needed to approach to find one AGYW | 5.42 | |
| # households needed to approach to find one AGYW interested in taking PrEP | 13.87 | |
| # households needed to approach to refer one AGYW for PrEP services | 14.08 | |
| # households needed to approach to initiate one AGYW on PrEP | 33.20 | |
